# Mesoionic N‐Heterocyclic Olefins, Imines, Thiones, Phosphinidenes and Their Application in Catalysis

**DOI:** 10.1002/anie.202520101

**Published:** 2025-11-29

**Authors:** Subir Maji, Sreejyothi P, Max M. Hansmann, Swadhin K. Mandal

**Affiliations:** ^1^ Department of Chemical Sciences Indian Institute of Science Education and Research Kolkata Mohanpur 741246 India; ^2^ Fakultät für Chemie und Chemische Biologie Technische Universität Dortmund Otto‐Hahn‐Str. 6 Dortmund 44227 Germany

**Keywords:** Mesoionic N‐heterocyclic imines, Mesoionic N‐heterocyclic olefins, Mesoionic N‐heterocyclic phosphinidenes, Mesoionic N‐heterocyclic thiones, Metal‐free catalysis

## Abstract

Transition metal compounds are widely known for their ability to activate small molecules and are excellent catalysts for a wide range of reactions. However, environmentally friendly and cost‐effective alternatives are highly desirable due to their toxicity and low abundance. In recent years, there has been a significant surge in the study of main‐group‐based species, mainly p‐block element‐derived compounds, which revealed reactivity resembling that of transition metal complexes. Recently, the mesoionic N‐heterocyclic carbenes (mNHCs) adducts of p‐block elements, containing exocyclic fragments such as ═CH_2_ (mNHOs), ═NR (mNHIs), ═PR (mNHPs), and ═S (mNHTs), have evolved as ligands for coordination chemistry, reactive species for the activation of small molecules, as well as efficient metal‐free catalysts for various challenging organic transformations, driven by their strong nucleophilicity. This minireview documents recent breakthroughs in the synthesis, reactivity, and catalytic applications of mNHOs, mNHIs, mNHTs, and mNHPs.

## Graphical abstract

This minireview covers recent advances in the synthesis, reactivity, and metal‐free catalytic uses of mesoionic N‐heterocyclic olefins (mNHOs), imines (mNHIs), thiones (mNHTs), and phosphinidenes (mNHPs).

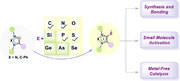

## 1 Introduction

**Scheme 1:**
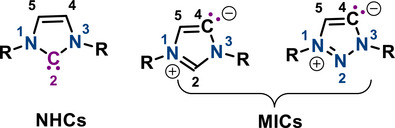
Schematic representation of NHCs and MICs.

Since the pioneering isolation of stable carbenes by Bertrand^[^ ^1^ ^]^ and N‐heterocyclic carbenes (NHCs) later by Arduengo,^[^ ^2^ ^]^ transforming a laboratory curiosity into a chemical reality, there has been tremendous growth in the development of NHCs and their associated chemistry.^[^ ^3^, ^4^, ^5^ ^]^ Their tuneable nucleophilicity and steric properties make them one of the most utilized scaffolds in organometallic chemistry and catalysis. Mesoionic carbenes (MICs) have emerged over the past two decades as a remarkable subclass of N‐heterocyclic carbenes (NHCs). Unlike classical NHCs, MICs exhibit canonical charge distributions that cannot be adequately represented by a single resonance structure, and they possess both positive and negative charges delocalized across the ring system and are hence called meso‐ionic (Scheme 1).^[^ ^6^ ^]^

The discovery of MICs was part of a broader wave of research that began with Arduengo's isolation of ‘normal’ N‐heterocyclic carbenes. The widespread use of NHCs as alternatives to phosphines in transition metal chemistry, combined with their rapid development, has resulted in the formation of a diverse library of carbenes.^[^ ^7^, ^8^ ^]^ Several years before the successful isolation of the first mesoionic carbene, Crabtree^[^ ^9^ ^]^ identified a unique bonding mode in which the carbene binds to the metal center through the C4 or C5 position of the heterocycle, rather than the typical carbene carbon adjacent to nitrogen (C2). This bonding mode was subsequently termed “abnormal” NHC coordination. Following the synthesis of the first “isolable” mesoionic carbene (MIC), imidazol‐5‐ylidene by Bertrand in 2009 (1,3‐bis(2,6‐diisopropylphenyl)‐2,4‐diphenyl‐imidazolylidene),^[^ ^10^ ^]^ the field of MIC chemistry experienced a rapid growth. This breakthrough revealed the unique electronic properties of MICs, characterized by their zwitterionic resonance structures and strong σ‐donor capabilities, which often surpass those of classical N‐heterocyclic carbenes (NHCs).^[^ ^11^, ^12^ ^]^ Soon after, the scope of MICs was expanded beyond imidazol‐5‐ylidenes to include a variety of heterocyclic frameworks such as pyrazolin‐4‐ylidene^[^ ^13^, ^14^ ^]^ and 1,2,3‐triazol‐5‐ylidenes.^[^ ^15^ ^]^

The development of such MIC scaffolds has enabled fine‐tuning of steric and electronic properties, thereby broadening their applicability in organometallic chemistry and metal‐free catalysis. Progressively, MICs have been incorporated into transition metal complexes,^[^ ^16^, ^17^ ^]^ demonstrating superior catalytic activity in cross‐coupling,^[^ ^18^ ^]^ olefin metathesis,^[^ ^19^ ^]^ and small‐molecule activation.^[^ ^20^ ^]^ More recently, advances in main‐group chemistry have utilized MICs for the stabilization of various otherwise unstable p‐block species, an area that is increasingly attracting significant attention.^[^ ^21^ ^]^

The unique distribution of charge in MICs, where the positive charge is delocalized across the ring structure and the negative charge is localized on the carbene center, results in very powerful σ‐donors. This high σ‐donating property makes them an ideal candidate for coordination with low‐valent main group elements.^[^ ^22^, ^23^, ^24^, ^25^, ^26^, ^27^ ^]^

The stabilization of low‐valent main‐group species by MICs occurs through a dual mechanism; the MIC lone pair donates into the empty orbital of the low‐valent center, stabilizing unoccupied low‐energy molecular orbitals. This interaction decreases reactivity and enhances thermodynamic stability.^[^ ^4^, ^21^, ^28^, ^29^, ^30^, ^31^ ^]^ Moreover, MICs are weak π‐acceptors and they do not participate significantly in π‐backbonding, and therefore do not withdraw electron density from the low‐valent center.^[^ ^32^ ^]^ In this way, MICs offer a great anchor for stabilizing and preserving the reactivity of p‐block elements.^[^ ^33^, ^34^ ^]^ This property makes the formal MIC–main‐group adducts particularly nucleophilic and unique in their electronic structure. Although the exploration of MIC‐stabilized main‐group compounds is still in its early stages, it is rapidly emerging as a vibrant new field with considerable potential for advancing main‐group chemistry.^[^ ^26^, ^35^ ^]^

**Scheme 2:**
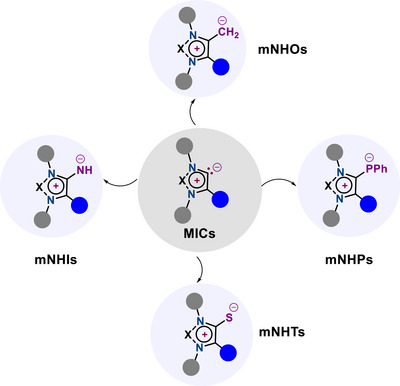
Formal p‐block element adducts of MICs (X ═ N, C─Ph).

MIC‐stabilized p‐block adducts reported to date include mesoionic carbene‐supported olefins (═CH_2_), imines (═NH), thiones (═S), as well as phosphinidenes (═P(I)‐R), which will be discussed in detail within this minireview (Scheme 2). It should be noted that the term “p‐block adducts”, as used throughout this review, refers to a formalism that differs from the classical/IUPAC definition of an “adduct”,^[^ ^36^ ^]^ but which is useful for systematically classifying the different compound classes in this review. Clearly, no cleavage of the C─C or C─N bond occurs in mesoionic N‐heterocyclic olefins (═CH_2_) and imines (═NR), respectively, despite what the word “adduct” might suggest. This review further discusses the reactivity of these formal adducts. Owing to their enhanced reactivity and distinct bonding characteristics, these adducts of MICs represent an emerging substance class in main group chemistry; however, a comprehensive review of this rapidly developing area is still lacking in the literature. In this minireview, we will focus on the p‐block adducts of MICs, their synthesis, bonding, reactivity, small molecule activation, and their use as metal‐free catalysts.

## 2 Mesoionic N‐Heterocyclic Olefins (mNHOs)

### 2.1 Synthesis and Reactivity

**Scheme 3:**
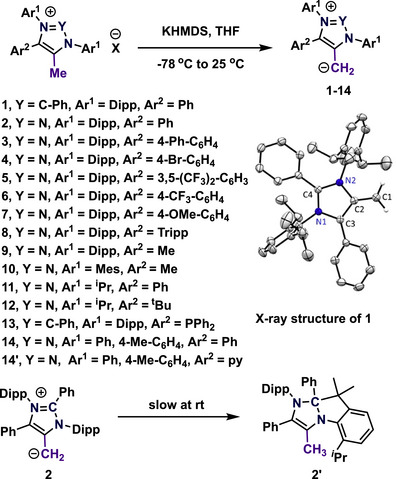
Top: Syntheses of mesoionic *N*‐heterocyclic olefins (mNHOs) and the solid‐state molecular structure of **1**. Adapted with permission from Ref. [37] © 2020, John Wiley and Sons. Bottom: Rearrangement of mNHO **2** to **2′**.(Dipp = 2,6 diisopropylphenyl, Mes = 1,3,5 trimethylphenyl, Tripp = 2,4,6‐triisopropylphenyl, KHMDS = Potassium hexamethyldisilazide).

Mesoionic N‐heterocyclic olefins (mNHOs) are alkylidene derivatives of mesoionic N‐heterocyclic carbenes.^[^ ^37^ ^]^ These compounds are neutral and feature a highly exocyclic polarized carbon‐carbon double bond, with the carbene position formally attached to a “CR_2_” group. Previously, there were very few mesoionic methylides described, mostly derived from 1,2,4‐triazole and tetrazole heterocycles; however, all of them featured strongly electron‐withdrawing groups (R ═ NO_2_, CN) at the exocyclic position.^[^ ^33^, ^38^, ^39^ ^]^ Such electron‐withdrawing groups strongly reduce the nucleophilic properties and do not allow specific reactivity, for instance in cases where the two H‐atoms are required for elimination. The mNHOs are typically prepared by deprotonating the corresponding imidazolium or 1,2,3‐triazolium salts (Scheme 3).^[^ ^40^ ^]^ Imidazolium salts are formed by reacting mesoionic N‐heterocyclic carbenes with methyl iodide, while 1,2,3‐triazolium salts are accessible via cycloaddition reactions.^[^ ^40^, ^41^, ^42^, ^43^ ^]^ In 2020, the Hansmann group was the first to report the synthesis and reactivity of mesoionic N‐heterocyclic olefins, including non‐stabilized systems with an exocyclic CH_2_ moiety (**1–2**, Scheme 3).^[^ ^37^ ^]^ The mNHOs– **1**–**2** were characterized using various spectroscopic methods as well as single‐crystal X‐ray crystallography (Scheme 3). Characteristic for the mNHOs is their unusual high‐field shifted ^1^H NMR [*δ*(^1^H) ∼ 24 ppm] and ^13^C NMR [*δ*(^13^C) ∼ 30–50 ppm] signals in agreement with their high electron density at the exocyclic moiety. The C1─C2 bond lengths in mNHOs (C1─C2 = 1.361–1.363 Å) are longer than those of typical heterocyclic olefins (NHOs; C1─C2 = 1.357 Å) and fall between single and double bonds, indicating mesoionic character.^[^ ^44^, ^45^ ^]^

**Scheme 4:**
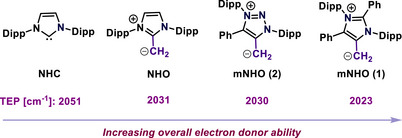
Overall donor ability of NHC, NHO, and mNHOs.

Interestingly, mNHO **1** remains stable in the solid‐state; however, it gradually undergoes rearrangement to produce the decomposition product of aNHCs (**2′**) at room temperature.^[^ ^10^ ^]^ Unlike NHOs, which are generally colorless solids, all the reported mesoionic olefins exhibit intense colors owing to their mesoionic nature.^[^ ^37^, ^46^, ^47^ ^]^ Later, Hansmann and coworkers reported various mNHOs (**3**–**12**) by altering different substituents, with a selection of these mNHOs being characterized through single‐crystal X‐ray diffraction (Scheme 3).^[^ ^48^, ^49^ ^]^ DFT calculations suggest that the lowest unoccupied molecular orbital (LUMO) is centered on the imidazole or triazole moiety, while the highest occupied molecular orbital (HOMO) and HOMO−1 are mainly on the exocyclic fragment.^[^ ^36^, ^48^, ^49^ ^]^ The overall donor capacity of mNHOs was examined utilizing the Tolman Electronic Parameter (TEP)^[^ ^50^ ^]^ by synthesizing mNHO‐rhodium carbonyl complexes and analyzing the IR‐spectroscopy of the carbonyl frequency. It may be noted that TEP represents the overall electron‐donating capability of a ligand; a lower value indicates a higher donor ability. It was observed that the overall donor ability of mNHOs (TEP: 2023 cm^−1^ (**1**); 2030 cm^−1^ (**2**)) significantly exceeded that of commonly reported ligand classes such as phosphines, N‐heterocyclic carbenes (NHCs; TEP = 2051 cm^−1^), mesoionic N‐heterocyclic carbene, and N‐heterocyclic olefins (NHOs; TEP = 2031 cm^−1^) (Scheme 4).^[^ ^37^ ^]^ Furthermore, Hansmann, Ofial, and colleagues reported the nucleophilicity of mNHOs (**5**–**12**) by examining the stopped‐flow kinetics of their reactions with arylidene malonates as reference electrophiles.^[^ ^49^ ^]^ Their findings demonstrated that the nucleophilicities could be extensively tuned (*N* = 20.8 to 31.9) through the modification of substituents. Alkyl‐substituted mNHOs (**11**–**12**) exhibit significantly higher nucleophilicity compared to their aryl‐substituted counterparts and are currently the strongest nucleophiles on the comprehensive Mayr nucleophilicity scale.^[^ ^51^, ^52^, ^53^ ^]^ The Mayr scale encompasses over 1000 nucleophiles involving C─, N─, O─, P─, S─, and Se‐centers, as well as nearly 200 hydride donors.^[^ ^51^ ^]^ They also studied the reactivity of mNHOs (**2**) for electrophilic fluorination and the CO_2_ fixation reaction.^[^ ^49^ ^]^

In 2024 the group of Bouffard reported a new synthesis route to mNHOs by the reaction of mesoionic carbenes (MICs) with N‐aziridinylimines.^[^ ^54^ ^]^ In 2022, the Ghadwal group documented the synthesis, characterization, and reactivity of a phosphine‐functionalized mNHO (**13**, Scheme 3).^[^ ^55^ ^]^ mNHO (**13**) was utilized for the stabilization of the dimeric gold complex [(**13**)_2_Au_2_]Cl_2_, [(**13**)_2_Au_2_]OTf_2_ and the boronium cation [(**13**)BPhCl]Cl.^[^ ^55^ ^]^ In 2024, the Kosmrlj group prepared the mNHO (**14**) coordinated palladium, gold, and boron hydride adducts. They also reported a pyridine tethered mNHO (**14'**), while the phosphine tethered failed upon deprotonation.^[^ ^56^ ^]^

**Scheme 5:**
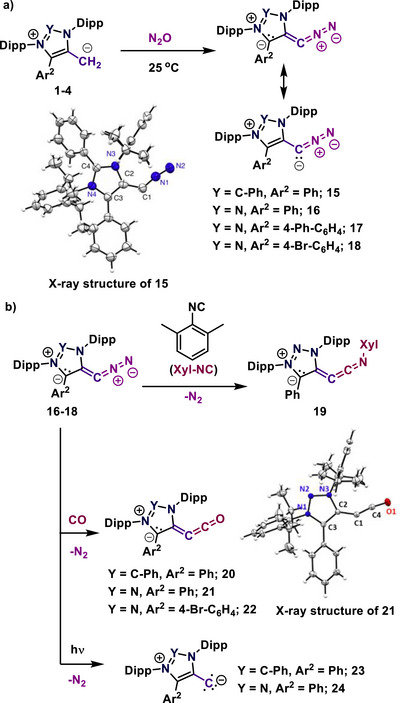
(a) Reactivity of mNHOs toward N_2_O for the syntheses of diazoalkenes and the solid‐state molecular structure of **15**, Adapted with permission from Ref. [57], © 2021, Springer Nature. (b) Reactivity of diazoalkenes with isocyanide and carbon monoxide, and solid‐state molecular structure of **21 (**Adapted with permission from Ref. [48], © 2021, American Chemical Society) irradiation of diazoalkenes to give triplet vinylidenes (**23**/**24**).

In 2021, the Hansmann group investigated how mNHOs (**1**–**4**) react with nitrous oxide (N_2_O).^[^ ^48^, ^57^ ^]^ This led to the formation of the first room‐temperature stable diazoalkenes (**15**–**18**) through diazo transfer reactions from N_2_O gas (Scheme 5). These mesoionic diazoalkenes are stable at room temperature under inert conditions, both in solid and solution states.^[^ ^58^ ^]^ They were characterized using spectroscopic methods and single‐crystal X‐ray crystallography in the solid‐state. ATR‐IR spectroscopy of the diazo group shows a distinct signal around 1944−1956 cm^−1^ (**15**–**18**), which is outside the typical range for organic diazo compounds (v∼ 2017−2180 cm^−1^).^[^ ^48^, ^57^ ^]^ This indicates considerable electron density at the CN_2_ moiety and significant π‐backdonation, which reduces the N─N bond order. The solid‐state structures showed that the C1─C2 bond lengths (1.391–1.401 Å) are intermediate, between single and double bonds, similar to the C─C bond in benzene. Additionally, the bond angle shows that diazoalkenes have a bent geometry. DFT calculations suggest that the lowest unoccupied molecular orbital (LUMO) is centered on the heterocycle, while the highest occupied molecular orbital (HOMO) and HOMO−1 are mainly on the CN_2_ fragment.^[^ ^48^, ^57^ ^]^ Further research was carried out to study the reactivity of mNHO‐derived diazoalkenes (**15**) toward different electrophiles and ambiphiles. Also, the displacement of N_2_ groups in the mNHO‐derived diazoalkenes **16**–**18** with an isocyanide and carbon monoxide (CO) at room temperature was examined, resulting in vinylidene ketenimines (**19**) and vinylidene ketenes (**20**–**22**), respectively, unambiguously characterized via X‐ray crystallography (Scheme 5).^[^ ^48^, ^59^, ^60^ ^]^ The N_2_/CO exchange mechanism entails a concerted process of nucleophilic activation of carbon monoxide, accompanied by the liberation of dinitrogen, as corroborated by the computational study of the potential energy surface.^[^ ^48^ ^]^ Interestingly, upon irradiation of the stable diazoalkenes, N_2_ can be liberated, allowing access to triplet vinylidenes **23** and **24**.^[^ ^61^, ^62^ ^]^ These compounds show a clear triplet ground state (ΔS/T > –9 kcal/mol) and could be characterized by EPR and ENDOR spectroscopy. They feature a zero‐field splitting parameter in the range of *D* ∼ 0.37–0.4 cm^−1^, a characteristic isotropic hyperfine coupling *A_iso_* (^13^C) ∼ 50 MHz, and a spin density of ca. 1.5 electrons on the exocyclic carbon atom. Depending on the substitution, this novel compound class can reach stabilities up to 150 K in toluene. Considering the relationship to mNHOs, the triplet vinylidenes are again mesoionic compounds, which feature a cationic heterocycle, and in the exocyclic position a negatively charged C‐atom. Very recently, phosphorus chelating mNHOs and chelating diazoalkenes have been reported by the Hansmann group.^[^ ^63^ ^]^

**Scheme 6:**
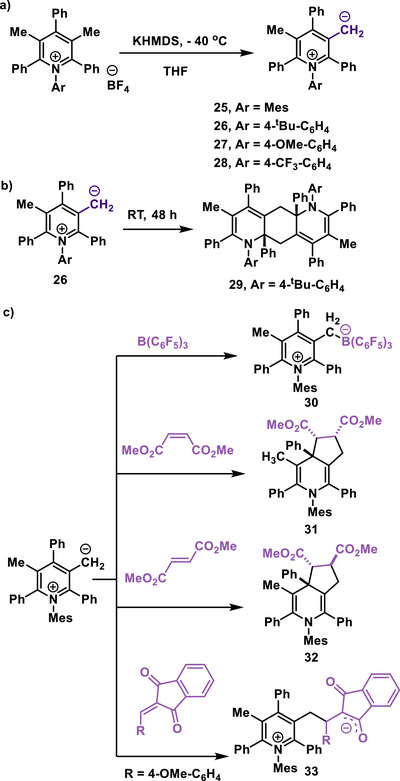
(a) Synthesis and reactivity of pyridinium‐derived mNHOs (py‐mNHOs). (b) Dimerization product of py‐mNHO (**26**). (c) Reactivity of py‐mNHOs.

In 2024, the Hansmann group reported the first pyridinium‐derived mesoionic N‐heterocyclic olefins (py‐mNHOs) (**25**–**28**).^[^ ^64^ ^]^ The py‐mNHOs were synthesized by deprotonation of the β‐CH_3_ group of a pyridinium salt with potassium bis(trimethylsilyl)amide (KHMDS) at low temperatures (Scheme 6a). These py‐mNHOs **25**–**28** were successfully isolated as deep green, crystalline solids by crystallizing the reaction mixture at –40 °C over 24 h (Scheme 6).^[^ ^64^ ^]^ The compounds **25**–**28** remain stable in the solid form at room temperature under inert conditions. However, keeping a solution of py‐mNHO **26** in benzene at room temperature for two days caused it to gradually form a dimer, designated as **29**, which is a formal (3 + 3) dimerization product, confirmed through X‐ray crystallography analysis (Scheme 6b).^[^ ^64^ ^]^ The formation of compound **29** occurred via nucleophilic attack at the electrophilic C2 position of the pyridine core.^[^ ^64^ ^]^

Furthermore, the reactivity of py‐mNHOs **25**–**28** with Lewis acids was examined. The reaction of **25** with the Lewis acid B(C_6_F_5_)_3_ produced the colorless adduct **30**, which was characterized by X‐ray analysis (Scheme 6c).^[^ ^64^ ^]^ The solid‐state structure confirmed the end‐on binding mode via the exocyclic methylene group to boron. Next, the use of py‐mNHOs in carbon–carbon bond‐forming reactions involving electron‐deficient π‐systems was explored (**31**–**32**). In contrast to the five‐membered mNHOs, py‐mNHOs show reactivity as 1,3‐dipoles. Two modes of 1,3‐dipolar cycloaddition are possible, forming five‐membered rings either across the exocyclic methylene group and the 4‐position or the 2‐position of the pyridinium core. Both reactions demonstrate high diastereoselectivity and can produce up to three adjacent stereocenters in a single step.^[^ ^64^ ^]^ Furthermore, the reaction of indandione methide with py‐mNHOs in toluene was studied, resulting in the formation of zwitterion **33**, where the indan‐1,3‐dione moiety effectively stabilizes the negative charge and the pyridinium moiety the cationic charge (Scheme 6c). The product was characterized by using both NMR spectroscopy and X‐ray diffraction analysis.^[^ ^64^ ^]^ Stopped‐flow techniques were used to determine the nucleophilicities of methyl and aryl‐substituted 2‐pyNHOs by following the kinetics of their reactions with reference electrophiles.^[^ ^65^ ^]^ The experimentally determined nucleophilicity values *N* in DMSO and acetonitrile are in the range of *N* = 19.4–21.2 (s_N_ ca. 0.6) on the Mayr reactivity scale, indicating significantly higher nucleophilic reactivity than observed for typical enamines such as 1‐(1‐phenylvinyl)piperidine, *N*,*N*‐dimethylcyclohex‐1‐en‐1‐amine, etc., which are used as organocatalysts.^[^ ^65^ ^]^

**Scheme 7:**
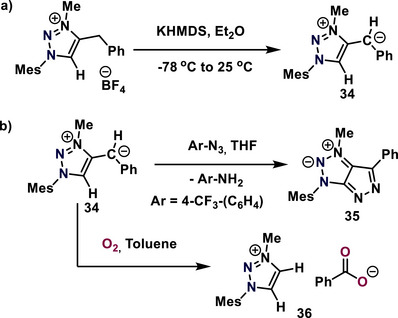
Synthesis and reactivity of the C‐5 unsubstituted mNHO **34**.

**Scheme 8:**
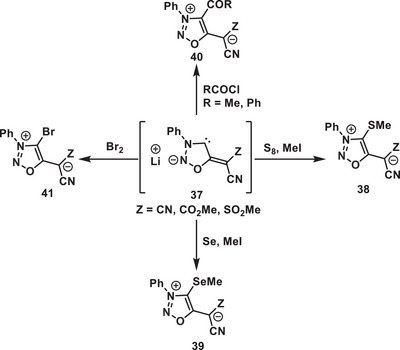
Examples of sydnone methides: a class of mNHOs.

The Song group reported that the C5‐unsubstituted mNHO (**34**) originate from 1,2,3‐triazolium featuring a benzyl group at the 4‐position, wherein the procarbenic (C5) site on the triazole ring remains unsubstituted (Scheme 7).^[^ ^66^ ^]^ Furthermore, the reactivity of mNHO **34** with Lewis acids such as 9‐BBN, BH_3_, and AlMe_3_ was investigated.^[^ ^66^, ^67^ ^]^ The presence of the electron‐rich ylidic olefin site alongside the relatively acidic proton at the procarbenic site in C5 unsubstituted mNHO can lead to distinctive reactivity behaviors. The C5‐unsubstituted mNHO **34** reacts with ArN_3_ to produce pyrazolo[3,4‐d][1,2,3]triazoles **35** and ArNH_2_ at room temperature (Scheme 7).^[^ ^68^ ^]^ The arylazide functions as a tandem diazo group transfer reagent and proton acceptor, facilitating the removal of olefinic and pro‐carbenic protons from the mNHO (**34**) to yield ArNH_2_. The mNHO **34** demonstrates unique reactivity toward O_2_, resulting in the formation of a triazolium benzoate salt **36** (Scheme 7).^[^ ^69^ ^]^ The mNHO which lacks vinyl protons or acidic C–H groups, is oxidized to form the corresponding triazolium oxide and ketones or aldehydes. In all instances, the initial C1─C2 bond of the mNHO (**34**) is cleaved.^[^ ^69^ ^]^ Recently, the Song research group reported the [4 + 1] cycloaddition of diazoester and mNHOs.^[^ ^70^ ^]^ The Schmidt group reported the synthesis of sydnone methides, a class of mNHOs (**38–41**) from **37**, where the positive charge is stabilized by the 1,2,3‐oxadiazolium ring and the negative charge is stabilized by exocyclic carbanionic groups like ‐C(CN)_2−_, ‐C(CN)(SO_2_Me)^−^, and ‐C(CN)(CO_2_Me) (Scheme 8).

### 2.2 Catalysis by mNHO

**Scheme 9:**
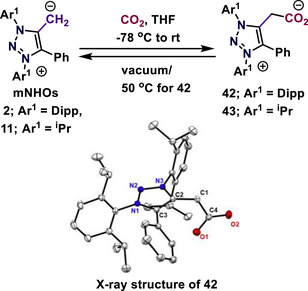
Reversible CO_2_ activation by mNHOs. Solid‐state molecular structure of **42**. Adapted with permission from Ref. [49], © 2023, John Wiley and Sons.

The Hansmann group demonstrated reversible CO_2_ activation using mNHOs.^[^ ^49^ ^]^ Since mNHOs are more nucleophilic than standard NHOs, it was expected that they would produce similar adducts with CO_2_.^[^ ^73^ ^]^ This prediction was confirmed by the observation that the N‐aryl and N‐alkyl substituted mNHOs **2** and **11**, respectively, produced the zwitterionic triazolium carboxylates **42** and **43** in good yields (Scheme 9).^[^ ^49^ ^]^ The new C─C bond in **42** (N‐Dipp) appeared to be weak, and heating at 50 °C, along with applying vacuum, resulted in the loss of CO_2_ and the regeneration of the mNHOs, while the more nucleophilic mNHO **11** formed a stable adduct. As a result, the more labile adduct **42** was dried under a stream of CO_2_. Crystals (**42**) suitable for single‐crystal X‐ray analysis were obtained under a CO_2_ atmosphere.^[^ ^49^ ^]^

**Scheme 10:**
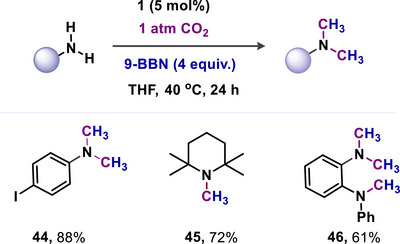
mNHO catalyzed *N*‐methylation of amines using CO_2_.

In 2021, Mandal and coworkers introduced mNHO (**1**)^[^ ^37^ ^]^ as a metal‐free catalyst for the reductive functionalization of CO_2_, facilitating consecutive double *N*‐methylation of primary amines with 9‐Borabicyclo(3.3.1)nonane (9‐BBN) serving as the hydride source under mild conditions (Scheme 10).^[^ ^74^ ^]^ This study explored the use of mNHO (**1**), known for its high nucleophilicity, to overcome the challenges of CO_2_ activation and functionalization due to its high kinetic and thermodynamic stability.^[^ ^75^, ^76^, ^77^ ^]^ The goal was to facilitate borane‐assisted CO_2_ reductive functionalization for *N*‐methylation of amines (**44–46**, Scheme 10).^[^ ^74^ ^]^ The high nucleophilicity of mNHO allowed the reactions to proceed more efficiently under milder conditions. The method successfully methylated a wide range of secondary and primary amines.^[^ ^74^ ^]^ The catalyst retained its activity through more than six consecutive N‐methylation cycles of secondary amines without any loss of performance.

Additionally, this catalytic N‐methylation reaction has been effectively utilized in the synthesis of pharmaceuticals using CO_2_. Overall, the research demonstrated that mNHO (**1**) functions as an effective catalyst for a 12‐electron reduction process, resulting in a double N‐methylation reaction (Scheme 10).^[^ ^74^ ^]^

**Scheme 11:**
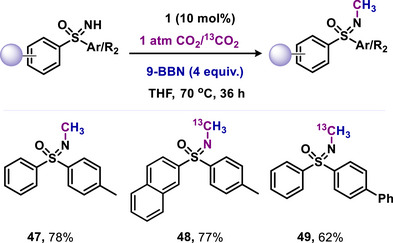
mNHO‐catalyzed *N*‐methylation of *N*H‐sulfoximines using CO_2_.

**Scheme 12:**
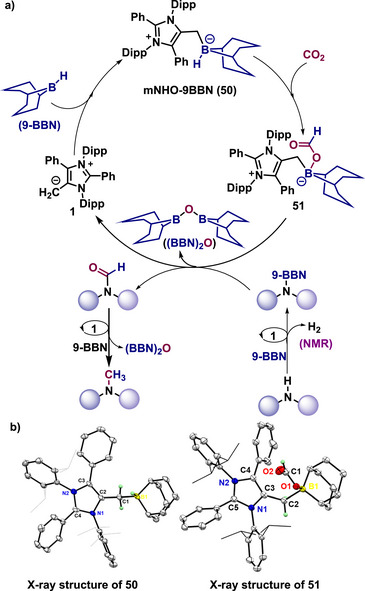
a) Possible mechanism for the mNHO‐catalyzed *N*‐methylation of amines and *N*H‐sulfoximines using CO_2_. b) X‐ray solid‐state molecular structure of the reaction intermediate mNHO‐9‐BBN adduct (**50,** Adapted with permission from Ref. [74], © 2021, Royal Society of Chemistry.) and boron formate (**51,** Adapted with permission from Ref. [78], © 2025, American Chemical Society).

Later, Mandal and coworkers extended this protocol to facilitate N‐methylation of NH‐sulfoximines with the use of CO_2_ (**47–49**, Scheme 11).^[^ ^78^ ^]^ The N‐methylation of sulfoximines with CO_2_ presents greater challenges compared to the methylation of amines, since NH‐sulfoximines are weaker bases and exhibit lower reactivity.^[^ ^79^, ^80^ ^]^ Consequently, functionalizing NH‐sulfoximines proves to be difficult due to the diminished nucleophilicity of the nitrogen atom. The high nucleophilicity of mNHO (**1**) facilitates the activation of less reactive NH‐sulfoximines.^[^ ^37^ ^]^ This protocol has been successfully employed for the catalytic N‐methylation of a broad spectrum of NH‐sulfoximines with CO_2_ in the presence of 9‐BBN, under metal‐free conditions (Scheme 11). Furthermore, it is applicable for the synthesis of ^13^C‐labelled N‐methyl sulfoximines utilizing ^13^CO_2_ in the reaction conditions (Scheme 11).^[^ ^78^ ^]^ Mechanistic studies on both N‐methylation protocols revealed a similar reaction pathway (Scheme 12).^[^ ^74^, ^78^ ^]^ A series of control experiments was conducted to clarify this mechanism, involving the isolation and characterization of various intermediates using single‐crystal X‐ray analysis or NMR spectroscopy for the mNHO‐catalyzed process. Initially, the control reactions showed that mNHO **1** reacts with 9‐BBN, forming the mNHO‐9‐BBN adduct **50** (Scheme 12). In this step, the borane's B─H bond is activated by coordination with the nucleophile mNHO **1**.^[^ ^74^ ^]^ The activated B─H bond then inserts into CO_2_, producing a boron formate intermediate **51** that was verified via single‐crystal X‐ray analysis.^[^ ^78^ ^]^ Simultaneously, the N─H bond reacts with 9‐BBN, creating the N‐borylated compound and releasing hydrogen gas (Scheme 12). Subsequently, the formyl group transfers from boron formate to the N‐borylated amine, resulting in N‐formyl amine and (9‐BBN)_2_O as a byproduct. After forming the N‐formylated product, it undergoes further reduction with 9‐BBN and the mNHO catalyst, leading to the N‐methylation product (Scheme 12).^[^ ^74^, ^78^ ^]^

**Scheme 13:**
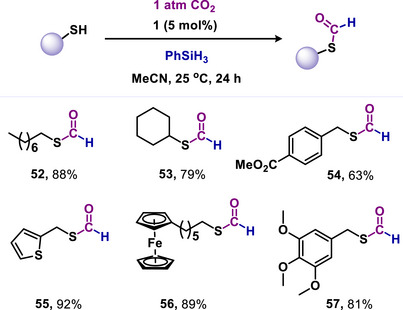
mNHO‐catalyzed *S*‐formylation of thiols using CO_2_.

In 2024, Mandal and coworkers introduced mNHO (**1**) as a metal‐free catalyst for the S‐formylation of thiols with CO_2_ (Scheme 13).^[^ ^81^ ^]^ It is important to emphasize that S‐formylated thiols and S‐formylation play vital roles in various biological processes, such as S‐formylglutathione and formyl coenzyme A are natural enzymes found in various living organisms.^[^ ^82^, ^83^, ^84^, ^85^ ^]^ Until this report, no catalytic chemical method was available to perform this key biological transformation using CO_2_. The reported protocol proved highly effective across a diverse range of thiols and dithiols, including benzylic, aliphatic, and bioactive types, some derived from clinically relevant drugs, under metal‐free conditions (**52–57**, Scheme 13).^[^ ^81^ ^]^ Additionally, this method was successfully applied for chemoselective S‐formylation in the presence of reducible functional groups.

**Scheme 14:**
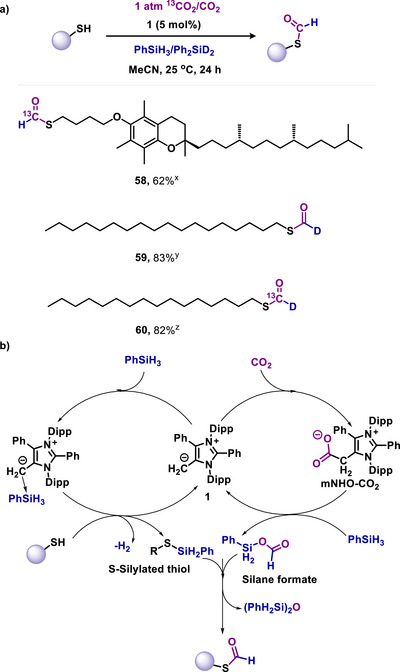
a) mNHO‐catalyzed *S*‐formylation of thiols using ^13^CO_2_/Ph_2_SiD_2_. ^x^PhSiH_3_ and ^13^CO_2,_^y^Ph_2_SiD_2_ and CO_2,_ Ph_2_SiD_2_ and ^13^CO_2_. b) Possible mechanism for the mNHO‐catalyzed S‐formylation of thiol using CO_2_.

**Scheme 15:**
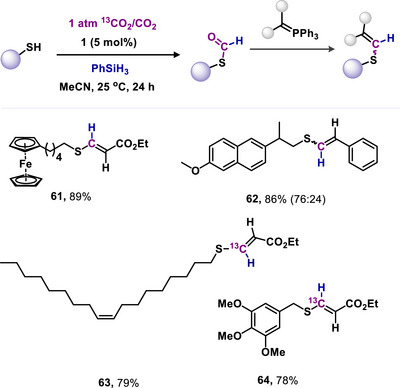
mNHO‐catalyzed one‐pot *S*‐formylation‐olefination of thiols using CO_2_/^13^CO_2_.

Furthermore, the S‐formylation process was extended for synthesizing a broad range of ^13^C‐labelled and deuterated formyl thiols (**58–60**, Scheme 14a). It should be noted that the incorporation of ^13^C labels and deuterium isotopes is commonly used in studying drug metabolism and related research on drug efficiency, pharmacokinetics, bioavailability, and tolerability. This method was also employed to synthesize the ^13^C‐labelled formyl coenzyme A.^[^ ^81^ ^]^ This intriguing transformation's mechanistic pathway was examined through multiple control experiments and the identification of key intermediates using spectroscopic and crystallographic techniques.^[^ ^81^ ^]^ The initial stage of the S‐formylation of thiols involves the activation of CO_2_. Here, mNHO (**1**) captures CO_2_, forming an mNHO‐CO_2_ adduct (Scheme 14b). This adduct then reacts with phenyl silane to form silyl formate. Simultaneously, mNHO (**1**) catalyzes the activation of the thiol with phenyl silane, resulting in S‐silylated thiol (Scheme 14b). Subsequently, this S‐silylated thiol reacts with silyl formate, transferring the formyl group and yielding the S‐formylated thiol (Scheme 14b).^[^ ^81^ ^]^ Additionally, this S‐formylation of thiols was employed to establish a one‐pot S‐formylation‐olefination method for synthesizing various internal olefins within the vinyl sulfides class (**61–64**, Scheme 15). One carbon atom in these internal olefins is directly sourced from CO_2_ under mild, metal‐free conditions. This work further demonstrated that the one‐pot olefination process is applicable for ^13^C labelling of these internal olefins with ^13^CO_2_. Consequently, this technique can be adapted to produce internal olefins from greenhouse gases, facilitating the synthesis of numerous vinyl sulfides from thiols and CO_2_ or ^13^CO_2_ (Scheme 15).^[^ ^81^ ^]^

**Scheme 16:**
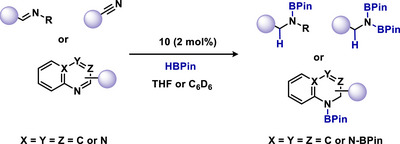
mNHO‐catalyzed hydroboration of imines, nitriles, and heteroarenes.

Yan and colleagues have reported that mNHO **10** demonstrates efficient catalytic activity in the hydroboration of aldimines, nitriles, and N‐heteroarenes using HBpin (Scheme 16).^[^ ^86^ ^]^ Compared to NHO (IprCH_2_; 1,3‐bis(2,6‐diisopropylphenyl)‐2‐methylene‐2,3‐dihydro‐1H‐imidazole), mNHO **10** provides higher yields even at reduced catalyst loadings. This improved performance is presumably due to the greater electron‐donating capacity of mNHO, which enhances the hydridic character of the borane hydride upon complexation.^[^ ^86^ ^]^

**Scheme 17:**
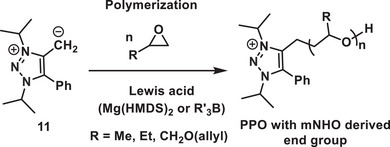
mNHO as initiators for epoxide polymerization (PPO; poly(propylene oxide), R = ‐Me, ‐Et and ‐CH_2_O (allyl)).

Recently, Hansmann, Naumann, and coworkers reported mNHO as initiators in conjunction with Lewis acidic catalysts for the polymerization of epoxides (Scheme 17).^[^ ^87^ ^]^ By employing mNHO (**11**)/Mg(HMDS)_2_ pairs, it was demonstrated that high‐yielding and rapid consumption of poly(propylene oxide) can be achieved. Furthermore, it was shown that mNHO initiators integrate effectively with borane‐type Lewis acids, which may be considered a milder alternative to Mg(HMDS)_2_.^[^ ^87^ ^]^ Overall, the obtained molar masses are up to two orders of magnitude lower than those observed with regular NHO/Mg(HMDS)_2_ pairs, reflecting the enhanced initiation efficiency attributable to the increased nucleophilicity of mNHO.^[^ ^87^ ^]^

**Scheme 18:**
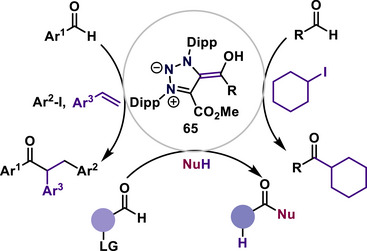
Breslow mNHO **65** as a super electron donor for various organic transformations.

Yan, Bertrand, and coworkers developed a mesoionic Breslow compound, a class of mNHO (**65**) bearing an exocyclic enol fragment, as a reaction intermediate for various metal‐free catalytic transformations, such as formyl alkylation of aldehydes, arylacylation of alkenes, and distal difunctionalization of aldehydes (Scheme 18).^[^ ^88^, ^89^, ^90^, ^91^, ^92^ ^]^ Such a type of functionalization is observed because of the single‐electron reduction by MIC‐deprotonated Breslow intermediate (**65**), which can serve due to its highly electron‐rich nature as an organic super electron donor.

## 3 Mesoionic N‐Heterocyclic Imines (mNHIs)

### 3.1 Synthesis and Reactivity

**Scheme 19:**
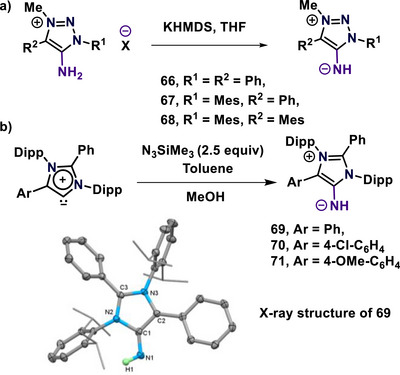
Syntheses of mNHIs and the solid‐state molecular structure of **69**. Adapted with permission from Ref. [94], © 2022, John Wiley and Sons.

The imine derivatives of mNHOs are known as mesoionic N‐heterocyclic imines (mNHIs). These contain a highly polarized exocyclic carbon‐nitrogen bond, where the carbene position is attached to an “NR” group. The mNHIs were synthesized via various methodologies.^[^ ^93^, ^94^, ^95^, ^96^, ^97^ ^]^ In 2022, Sarkar and colleagues reported the synthesis of mNHIs (**66**–**68**) through the deprotonation of the corresponding triazolium salt employing KHMDS (Scheme 19).^[^ ^93^ ^]^ Subsequently, the Mandal group reported the synthesis of mNHIs from mesoionic N‐heterocyclic carbenes via a Staudinger‐type reaction (Scheme 19).^[^ ^94^ ^]^

**Scheme 20:**
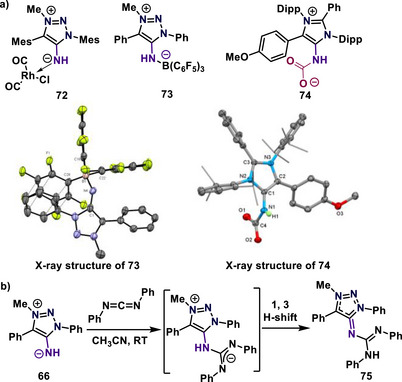
a) Reactivity of mNHIs with CO_2_, B(C_6_F_5_)_3,_ and [Rh(CO)_2_Cl]_2_ and solid‐state molecular structures of **73 (**Adapted with permission from Ref. [93], © 2022, John Wiley and Sons). and **74**. Adapted with permission from Ref. [94], © 2022, John Wiley and Sons. b) Synthesis of mesoionic N‐heterocyclic imine‐carbodiimide (mNHI–CDI) adduct (**75**).

The toluene solution of aNHCs was treated with azidotrimethylsilane, and the reaction mixture was refluxed for 60 h.^[^ ^98^ ^]^ Upon solvent removal, the residue was treated with methanol, resulting in mNHIs (**69**–**71**) as bright orange solids (Scheme 19).^[^ ^94^ ^]^ They were characterized using spectroscopic methods and single‐crystal X‐ray crystallography (Scheme 19). The C1─N1 bond lengths range from 1.299 to 1.306 Å, slightly longer than a typical C─N double bond. These mesoionic N‐heterocyclic imines (**69**–**71**) remained stable under an argon atmosphere at room temperature.^[^ ^94^ ^]^ The bond lengthening supports their mesoionic nature, a key characteristic of these molecules. Unlike conventional N‐heterocyclic imines (NHIs), which are generally colorless, mNHIs display a striking yellow to orange color due to HOMO‐LUMO charge transfer transitions from the negatively charged ‐NH group to the cationic imidazole or triazole moiety.^[^ ^93^, ^94^ ^]^ They demonstrate prominent absorption bands within the visible spectrum at 518–526 nm. These features correlate with electronic transitions between the highest occupied molecular orbital (HOMO) and the lowest unoccupied molecular orbital (LUMO), involving charge transfer from the negatively polarized ‐NH group to the cationic N‐heterocyclic core. This charge movement accounts for the vivid coloration of the mNHIs and was confirmed by computational TD‐DFT studies, which predicted absorption maxima close to the experimental values.^[^ ^94^ ^]^ Further analysis demonstrated that the proton affinities (PAs) for mNHIs are among the highest documented for N‐heterocyclic imines.^[^ ^99^, ^100^, ^101^, ^102^ ^]^ The PA values for mNHIs **69**–**71** range from 278.5 to 283.7 kcal/mol, respectively, surpassing the triazole‐mNHIs **66**–**68** (∼271.4 kcal/mol). These donor characteristics of mNHIs were investigated through their reactivity with transition metals (**72**; Ir, Rh) and Lewis acids (**73**; B(C_6_F_5_)_3_, **74**; CO_2_, Scheme 20a).^[^ ^93^, ^94^ ^]^ Later, Sarkar and colleagues reported various transition metal complexes stabilized by mNHIs, along with their catalytic activity in the hydrogen transfer reaction of ketones in the presence of isopropanol.^[^ ^103^, ^104^, ^105^ ^]^

Recently, Sarkar and coworkers reported the synthesis of triazole based mesoionic N‐heterocyclic imine‐carbodiimide (mNHI‐CDI) adduct (**75**, Scheme 20b).^[^ ^106^ ^]^ The Tolman Electronic Parameter (TEP) for compound **69** was measured at 2033 cm^−1^, indicating that mNHIs are superior donors compared to conventional NHIs and triazole‐mNHIs, which exhibit TEP values ranging from 2041 cm^−1^ to 2044 cm^−1^. The lower TEP values further confirmed the strong donor ability of mNHIs (**69**) and their promising utility in catalytic applications.^[^ ^93^, ^94^ ^]^ In this context, Mandal and colleagues successfully synthesized and elucidated the structure of a mNHI‐CO_2_ adduct (**74**) via X‐ray crystallography (Scheme 20a).^[^ ^94^ ^]^ The molecular structure of mNHI‐CO_2_ adduct **74** reveals that the N1─C1 bond length has considerably increased to 1.379 Å from 1.3063 Å (in compound **69**) following coordination with CO_2_.^[^ ^94^ ^]^ Notably, mNHIs exhibit reversible CO_2_ activation upon exposure to heat or vacuum.^[^ ^93^, ^94^ ^]^

### 3.2 Metal‐Free Catalysis by mNHI

**Scheme 21:**
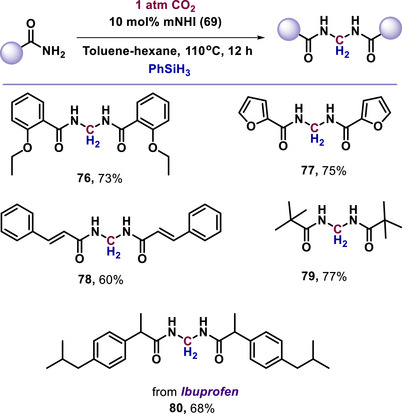
mNHI‐catalyzed coupling of amides by deoxygenation of CO_2_.

The reversible CO_2_ activation property of mNHIs was adapted to catalyze the conversion of CO_2_ into value‐added products. Mandal and colleagues demonstrated the catalytic ability of mNHI (**69**) to facilitate amide coupling through the deoxygenation of CO_2_ in the presence of phenylsilane under reductive conditions (**76–80**, Scheme 21).^[^ ^94^ ^]^

**Scheme 22:**
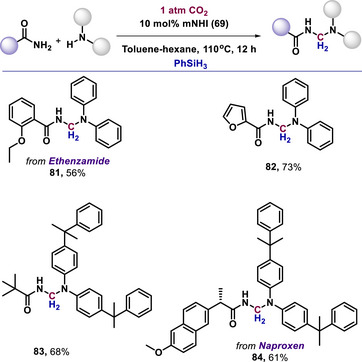
mNHI‐catalyzed hetero coupling of amines and amides using CO_2_.

Furthermore, mNHI (**69**) enabled the coupling of amides with amines via the deoxygenation of CO_2_ under reductive conditions (**81–84**, Scheme 22).^[^ ^94^ ^]^ This documented protocol was also applicable to a diverse range of amides and amines for the synthesis of value‐added chemicals by utilizing CO_2_.

The mechanistic pathway for the methylenation reaction was further clarified through a series of control experiments.^[^ ^94^ ^]^ Initially, mNHI captures CO_2_ to form the mNHI‐CO_2_ adduct. This adduct then reacts with phenylsilane to form silane formate. Also, mNHIs catalyzed the activation of amides to N‐silyl amides in the presence of phenylsilane. Then, N‐silyl amide reacts with silane formate to form an N‐formyl amide, which subsequently undergoes hydride transfer to generate the final methylene‐linked coupling product.^[^ ^94^ ^]^

**Scheme 23:**
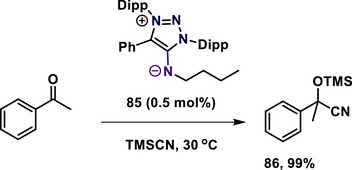
mNHI‐catalyzed cyanosilylation of acetophenone.

Haraguchi and coworkers reported the mesoionic N‐heterocyclic imine **85** as a metal‐free catalyst for the cyanosilylation of acetophenone, which afforded the TMS protected cyanohydrin **86** under mild conditions (Scheme 23).^[^ ^95^, ^97^ ^]^

## 4 Mesoionic N‐Heterocyclic Thiones (mNHTs)

### 4.1 Synthesis and Reactivity

**Scheme 24:**
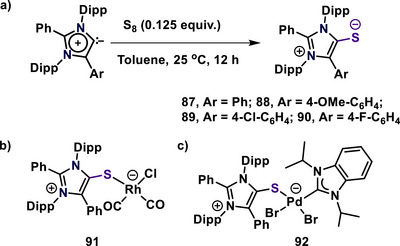
(a) Synthesis of mesoionic N‐heterocyclic thiones (mNHTs, **87**–**90**). (b) mNHT‐rhodium complex (**91)**. (c) mNHT‐palladium complex (**92**).

Mesoionic N‐heterocyclic thiones (mNHTs) are sulfur derivatives of mesoionic N‐heterocyclic carbenes. These contain a highly polarized exocyclic carbon‐sulfur bond, where the carbene position is attached to an “S”. The mNHTs were synthesized via various methodologies.^[^ ^107^, ^108^, ^109^, ^110^ ^]^ Recently, Mandal and colleagues reported the synthesis and reactivity of mNHTs derived from mesoionic N‐heterocyclic carbenes.^[^ ^110^ ^]^ These mNHTs (**87**–**90**) were synthesized through the reaction of abnormal N‐heterocyclic carbenes (aNHCs) with elemental sulfur in toluene at ambient temperature (Scheme 24).^[^ ^110^ ^]^ Notably, mNHT exhibits high stability under atmospheric conditions, preserving their structural integrity for several months in both solid and solution states. Conversely, MICsmNHOs, andmNHIs, undergo rapid decomposition when exposed to air. DFT calculations showed that the HOMO is localized on sulfur, with the LUMO being delocalized over the imidazolium ring. A charge of −0.35e on sulfur suggests polarity in the C1─S1 bond. Bond analysis indicates a bond order of 1.33, implying the C1─S1 bond is intermediate between a single and a double bond. Natural bonding orbital analysis indicates dual‐site reactivity: sulfur as nucleophile and the imidazolium heterocycle as an electrophile.^[^ ^110^ ^]^

Fukui calculations of mNHT **87** confirm sulfur's nucleophilicity and the imidazole ring's electrophilicity. To evaluate the mesoionic N‐heterocyclic thione's donor ability, complex **91** was synthesized, exhibiting a TEP of 2038 cm^−1^, which is smaller than that of previous NHTs (2047–2049 cm^−1^), indicating a higher donor ability of mNHTs than NHTs (Scheme 24). Also, the Huynh Electronic Parameter (HEP; A method to evaluate the ligand donor ability using ^13^C NMR spectroscopy of the palladium NHC complex, was determined by synthesizing the mNHT palladium complex **92**, suggesting the moderate donor ability in the Huynh scale (Scheme 24).^[^ ^111^ ^]^ The reactivity of **87** with electrophiles, including boron and bismuth electrophiles, afforded various mNHT (**87**) stabilized main‐group compounds, which were confirmed by X‐ray diffraction.^[^ ^110^ ^]^

### 4.2 mNHTs‐Derived Radical Anion and Its Reactivity

**Scheme 25:**
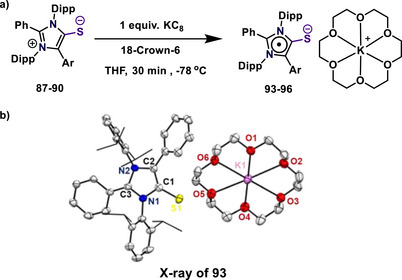
(a) Synthesis of mesoionic *N*‐heterocyclic thiones derived radical anions. (b) Solid‐state molecular structure of **93**. Adapted with permission from Ref. [110], © 2024, John Wiley and Sons.

Cyclic voltammetry of mNHTs revealed two reversible one‐electron reductions (*E*_1/2_: 1.30 V; −1.44 V and 1.80 V; −1.95 V (versus Ag/Ag^+^), demonstrating their electron‐accepting character. Radical anions (**93**–**96**), synthesized by the reduction of mNHTs with KC_8_, were characterized by EPR spectroscopy, which showed hyperfine interactions with the nitrogen atoms and the phenyl hydrogen atoms supported by computational data (Scheme 25).^[^ ^110^ ^]^ X‐ray crystallography confirmed the structure of radical anion **93**, indicating minimal bond changes in the thione group but elongation in N1─C3 and C3─N2 bonds, implying electron localization on the imidazole ring (Scheme 25). Spin density and molecular orbital analyses revealed that the LUMO of **87** becomes the SOMO of **93** after reduction, with an energy increase of 1.11 eV. Fukui analysis identified the imidazole ring as the primary site of the LUMO.

**Scheme 26:**
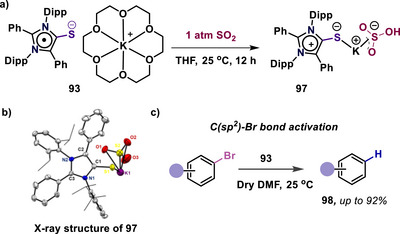
(a) Reactivity of radical anion (**93**) for the activation of SO_2_ gas. (b) Solid‐state molecular structure of **97**. Adapted with permission from Ref. [110], © 2024, John Wiley and Sons. (c) Reactivity of radical anion (**93**) for the activation of a C(sp^2^)‐Br bond.

Furthermore, the radical anion **93** was utilized for the activation of SO_2_ gas through a single‐electron transfer process, and its structure (**97**) was characterized by single‐crystal X‐ray diffraction. Also, the radical anion has been applied for the stoichiometric dehalogenation of aryl bromides to the corresponding arenes (**98**) (Scheme 26).^[^ ^110^ ^]^

### 4.3 Metal‐Free Catalysis by mNHT

**Scheme 27:**
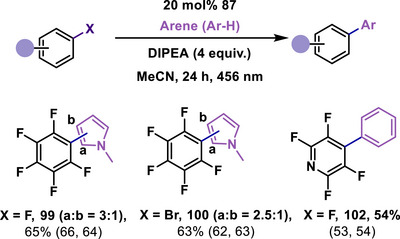
Mesoionic N‐heterocyclic thione (mNHT, **87**) as a photocatalyst for the catalytic C(sp^2^)‐X (X = F, Br) bond functionalization of aryl halides to access biaryl polyfluorinated compounds.

The mNHT **87** was investigated as a metal‐free photocatalyst for activating C─F bonds in polyfluorinated compounds, resulting in the synthesis of multifluorinated biaryl compounds (**99–102**, Scheme 27).^[^ ^110^ ^]^

## 5 Mesoionic N‐Heterocyclic Phosphinidenes (mNHPs)

### 5.1 Synthesis and Reactivity

**Scheme 28:**
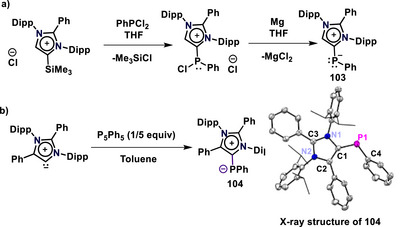
(a) Synthesis of a mesoionic N‐heterocyclic phosphinidene by reduction of phosphorus precursor. (b) Synthesis of an abnormal N‐heterocyclic phosphinidene (**104**, Adapted with permission from Ref. [113], © 2025, American Chemical Society) from a mesoionic carbene.

Mesoionic N‐heterocyclic phosphinidenes (mNHPs) are low‐valent phosphorus compounds derived from mNHCs, in which the carbene carbon stabilizes a monovalent phosphorus center bearing two lone pairs. They are typically prepared either by transferring a phosphinidene fragment to the mesoionic carbene scaffold or by reduction of carbene‐anchored phosphorus precursors, yielding highly reactive, nucleophilic, and electronically tunable phosphorus centers.^[^ ^112^, ^113^ ^]^ Ghadwal and coworkers synthesized mNHP from C4‐silylated 1,3‐imidazolium salt (MIC‐SiMe_3_) as transfer agents.^[^ ^112^ ^]^ Treatment with PhPCl_2_ released Me_3_SiCl, affording the corresponding mNHP precursor. Subsequent reduction of this intermediate yielded the mNHP **103** in 56% isolated yield (Scheme 28a). The ^31^P{^1^H} NMR resonance of the compound appeared at δ = −48.2 ppm in benzene‐*d_6_*. Very recently, the Mandal group reported the synthesis of an mNHP from the isolated abnormal N‐heterocyclic carbene via treatment with pentaphenylcyclopentaphosphane (P_5_Ph_5_) in toluene.^[^ ^113^ ^]^ The corresponding phosphinidene **104** was isolated as a deep brown solid in 57% yield and fully characterized by X‐ray crystallography (Scheme 28b).

**Scheme 29:**
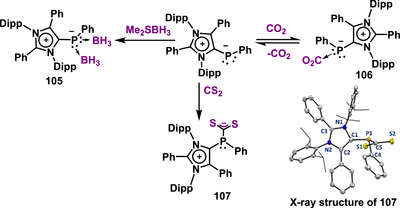
Nucleophilic reactivity of mesoionic N‐heterocyclic phosphinidene (mNHP) with various small molecules. Adapted with permission from Ref. [113], © 2025, American Chemical Society.

The ^31^P{^1^H} NMR resonance appeared at δ = −52.1 ppm (acetonitrile‐d_3_), consistent with a highly shielded phosphorus nucleus. X‐ray analysis revealed a bent (V‐shaped) geometry around the phosphorus center, with a C1─P1─C4 bond angle of 102.73(9)°, which is notably smaller than the ideal angle for an sp^3^‐hybridized phosphorus atom.^[^ ^114^ ^]^ The C1(carbene)–P1 bond length in **104** [1.803(2) Å], is only slightly shorter than the single P1─C4(phenyl) bond [1.819(2) Å], and significantly longer than the typical C═P bond distance in phosphaalkenes (1.65–1.67 Å),^[^ ^114^ ^]^ indicating that the C1–P1 bond is predominantly single‐bond in character. This was further supported by the Wiberg Bond Index (WBI) analysis, which gave a value of 1.12, the lowest among all the main‐group adducts discussed herein. The mNHP contains two lone pairs of electrons at the phosphorus center. To demonstrate that both are available for donation, an mNHP–bis(borane) adduct (**105**) was synthesized by treatment with two equiv. of BH_3_·SMe_2_ (Scheme 29), which was structurally confirmed by X‐ray crystallography. The nucleophilic nature of mNHP was further evaluated through small‐molecule activation. In the reaction with CO_2_, NMR spectroscopy revealed that a reversible mNHP–CO_2_ adduct (**106)** was formed that exists in equilibrium, releasing CO_2_ under vacuum and reforming the adduct upon re‐exposure to CO_2_ (Scheme 29). However, a stronger binding was observed when CS_2_ was used instead of CO_2_, leading to the successful synthesis of the mNHP–CS_2_ adduct (**107**) (Scheme 29), which was characterized by various spectroscopic techniques, including X‐ray crystallography. All these reactions confirmed the strong nucleophilic properties of mNHP.

### 5.2 Catalysis by P(I)/P(III) Redox Behavior of mNHP

**Scheme 30:**
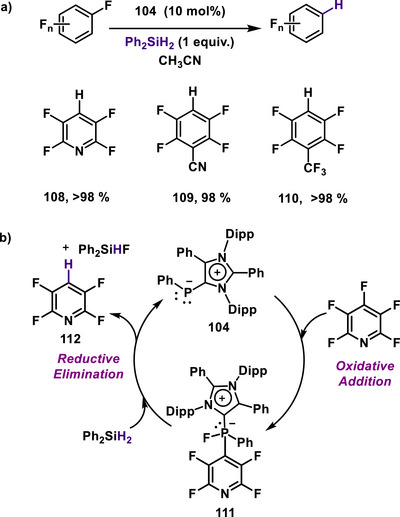
(a) mNHP catalyzed hydrodeflourination of fluoro‐arenes. (b) P(I)/P(III) redox catalytic cycle for hydrodefluorination.

Furthermore, the mesoionic N‐heterocyclic phosphinidene was employed as a transition metal mimicking catalyst by redox switching between P(I)/P(III) species, demonstrating redox activity.^[^ ^10^ ^]^ Catalytic applications involve the activation of C─F bonds, establishing a P(I)/P(III) redox cycle that enables oxidative addition at the low‐valent phosphorus center. Notably, mNHP was applied in the catalytic hydrodefluorination of aryl fluorides, a transformation previously unknown for P(I)/P(III) redox catalysis. In the presence of 1 equiv. of Ph_2_SiH_2_, catalytic mNHP efficiently promoted the hydrodefluorination of various aryl fluorides to generate the corresponding arenes (Scheme 30a, **108**–**110**). This protocol was also successful when using deuterium‐labelled Ph_2_SiH_2_, affording deuterated arenes. Mechanistic studies revealed that the reaction proceeds via a typical transition metal–like pathway involving P(I)/P(III) redox catalysis. A stoichiometric reaction between mNHP and the activated aryl fluoride pentafluoropyridine demonstrated oxidative addition at the phosphorus center, in which fluorine was incorporated at the phosphorus center, generating a P(III) species, **111**. The oxidative addition product was characterized using various spectroscopic techniques, including X‐ray photoelectron spectroscopic analysis. The ^31^P{^1^H} NMR spectrum displayed a doublet at δ 9.0 ppm, a substantial downfield shift from −52 ppm, indicating increased coordination at the phosphorus nucleus, with a significant P–F coupling constant of 933 Hz. The ^1^⁹F{^1^H} NMR spectrum exhibited a doublet at δ −53.0 ppm with a similar coupling constant (929 Hz), further confirming the presence of a P─F bond. High‐resolution mass spectrometry (HRMS) also supported the proposed product structure. At this stage, in the presence of the hydride donor Ph_2_SiH_2_, a hydride transfer to the oxidative addition product occurs, leading to the release of the hydrodefluorinated product (**112**) and regeneration of mNHP, along with the formation of Ph_2_SiHF, thus completing the redox cycle (Scheme 30b).

## 6 Summary and Outlook

Over the past five years, remarkable progress has been made in developing p‐block element adducts of mesoionic N‐heterocyclic carbenes (MICs). This minireview highlights recent advances in the synthesis, reactivity, activation of small molecules, and metal‐free catalytic applications of such formal adducts, specifically mNHOs, mNHIs, mNHTs, and mNHPs. Such developments are reminiscent of the transition metal's ability to activate small molecules, followed by catalytic transformations. The strong σ‐donor ability and limited π‐acceptor nature of MICs allow the stabilization of highly nucleophilic p‐block adducts, surpassing their normal N‐heterocyclic carbene counterparts in donor strength. Among them, mNHOs exhibit the highest donor capacity and nucleophilicity, enabling efficient activation and functionalization of challenging small molecules such as N_2_O, CO_2_, and O_2_, with notable success in CO_2_ functionalization catalysis. While mNHOs are already well‐established in metal‐free catalysis, the development of mNHIs, mNHTs, and mNHPs remains comparatively nascent. Harnessing transition metal‐like features, such as P(I)/P(III) redox activity, could significantly broaden their catalytic scope, including transformations traditionally dominated by transition metals. There remains considerable room to explore and investigate the full potential of these molecules across various applications for instance in organocatalysis. Nevertheless, the progress observed so far suggests that this area could deliver many groundbreaking discoveries, owing to the presence of highly reactive centers in these compounds. Furthermore, vivid coloration of many p‐block MIC adducts also makes them promising candidates for photocatalysis, while analogous systems, such as MIC‐stabilized bismuthinidenes, may offer opportunities for exploring new reactivity. While questions may arise regarding the handling and sensitivity of these compounds, continued optimization and rational molecular design are likely to overcome these challenges. In this context, MIC‐stabilized main‐group adducts could emerge as viable alternatives to transition metals across a wide range of catalytic transformations. Looking ahead, MIC‐stabilized p‐block adducts hold substantial promise as metal‐free catalysts, not only for fine chemical synthesis but also for discovering reactions with inert small molecules.

## Conflict of Interests

The authors declare no conflict of interest.

## Data Availability

Data sharing is not applicable to this article as no new data were created or analyzed in this study.
